# (*Z*)-1-(2,4-Difluoro­phen­yl)-3-phenyl-2-(1*H*-1,2,4-triazol-1-yl)prop-2-en-1-one

**DOI:** 10.1107/S1600536809029821

**Published:** 2009-07-31

**Authors:** Cong-Yan Yan, Guang-Zhou Wang, Cheng-He Zhou

**Affiliations:** a Laboratory of Bioorganic & Medicinal Chemistry, School of Chemistry and Chemical Engineering, Southwest University, Chongqing, 400715, People’s Republic of China

## Abstract

In the title mol­ecule, C_17_H_11_F_2_N_3_O, the triazole ring makes dihedral angles of 83.00 (5) and 16.63 (5)°, respectively, with the phenyl and benzene rings. Weak inter­molecular C—H⋯F and C—H⋯N inter­actions contribute to the crystal packing.

## Related literature

For details of the synthesis, see: Wang *et al.* (2009 ▶). For the pharmacological activity of chalcones, see: Reichwald *et al.* (2008 ▶).
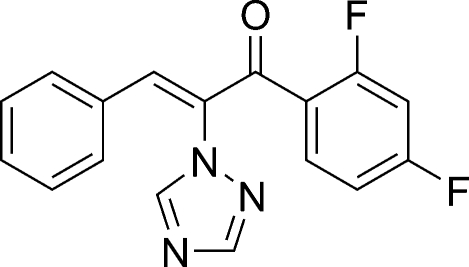


## Experimental

### 

#### Crystal data

C_17_H_11_F_2_N_3_O
*M* *_r_* = 311.29
Monoclinic, 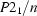

*a* = 11.7595 (16) Å
*b* = 7.5800 (10) Å
*c* = 17.068 (2) Å
β = 108.067 (2)°
*V* = 1446.4 (3) Å^3^
*Z* = 4
Mo *K*α radiation
μ = 0.11 mm^−1^
*T* = 296 K
0.25 × 0.21 × 0.14 mm

#### Data collection

Bruker SMART CCD area-detector diffractometer
Absorption correction: multi-scan (*SADABS*; Sheldrick, 1997 ▶) *T*_min_ = 0.973, *T*_max_ = 0.985
10616 measured reflections
2681 independent reflections
1940 reflections with *I* > 2σ(*I*)
*R*_int_ = 0.029

#### Refinement

*R*[*F* ^2^ > 2σ(*F* ^2^)] = 0.040
*wR*(*F* ^2^) = 0.101
*S* = 1.02
2681 reflections
208 parameters
H-atom parameters constrained
Δρ_max_ = 0.11 e Å^−3^
Δρ_min_ = −0.19 e Å^−3^

### 

Data collection: *SMART* (Bruker, 1997 ▶); cell refinement: *SAINT-Plus* (Bruker, 1997 ▶); data reduction: *SAINT-Plus*; program(s) used to solve structure: *SHELXS97* (Sheldrick, 2008 ▶); program(s) used to refine structure: *SHELXL97* (Sheldrick, 2008 ▶); molecular graphics: *SHELXTL* (Sheldrick, 2008 ▶); software used to prepare material for publication: *SHELXTL*.

## Supplementary Material

Crystal structure: contains datablocks I, global. DOI: 10.1107/S1600536809029821/cv2596sup1.cif

Structure factors: contains datablocks I. DOI: 10.1107/S1600536809029821/cv2596Isup2.hkl

Additional supplementary materials:  crystallographic information; 3D view; checkCIF report

## Figures and Tables

**Table 1:** Hydrogen-bond geometry (Å, °)

*D*—H⋯*A*	*D*—H	H⋯*A*	*D*⋯*A*	*D*—H⋯*A*
C12—H12⋯N3^i^	0.93	2.68	3.540 (2)	154
C17—H17⋯F1^ii^	0.93	2.62	3.280 (2)	128

Symmetry codes: (i) 
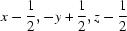
; (ii) 
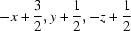
.

**Fig. 1.**
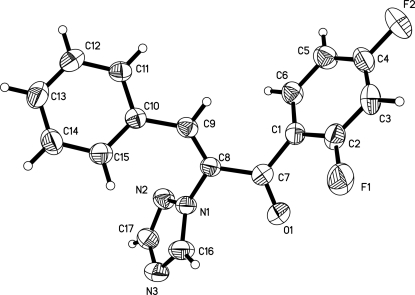
The molecular structure of (I), showing the atom-numbering scheme. Displacement ellipsoids are drawn at the 50% probability level.

## supplementary crystallographic information

### Comment

Chalcones, considered as the precursors of flavonoids and isoflavonoids, are abundant in edible plants. Chalcones exhibit a variety of beneficial pharmacological activities such as antitumor, antibacterial, antifungal, antiinflammatory, antimalarial, antivirus and so on (Reichwald *et al.*, 2008). In view of the therapeutic potentials of chalcones, we synthesized the title compound (I). Herewith we report its crystal structure.

In (I) (Fig. 1), the triazole ring makes the dihedral angles of 83.00 (5)° and 16.63 (5)°, respectively, with the phenyl and benzene rings. Weak intermolecular C—H···F and C—H···N interactions (Table 1) contribute to the crystal packing stability.

### Experimental

Compound (I) was synthesized according to the procedure of Wang *et al.* (2009). A crystal of (I) suitable for X-ray analysis was grown from a mixture solution of ethyl acetate and petroleum ether by slow evaporation at room temperature.

### Refinement

All the hydrogen atoms were placed at the geometrical positions with C—H = 0.93 Å, and refined as riding, with *U*iso(H) = 1.2 *U*eq(C).

### Crystal data

**Table:**

C_17_H_11_F_2_N_3_O	*F*(000) = 640
*M**_r_* = 311.29	*D*_x_ = 1.429 Mg m^−^^3^
Monoclinic, *P*2_1_/*n*	Mo *K*α radiation, λ = 0.71073 Å
*a* = 11.7595 (16) Å	Cell parameters from 2124 reflections
*b* = 7.580 (1) Å	θ = 2.5–23.7°
*c* = 17.068 (2) Å	µ = 0.11 mm^−^^1^
β = 108.067 (2)°	*T* = 296 K
*V* = 1446.4 (3) Å^3^	Block, colourless
*Z* = 4	0.25 × 0.21 × 0.14 mm

### Data collection

**Table:**

Bruker SMART CCD area-detector diffractometer	2681 independent reflections
Radiation source: fine-focus sealed tube	1940 reflections with *I* > 2σ(*I*)
graphite	*R*_int_ = 0.029
φ and ω scans	θ_max_ = 25.5°, θ_min_ = 2.5°
Absorption correction: multi-scan (*SADABS*; Sheldrick, 1997)	*h* = −14→14
*T*_min_ = 0.973, *T*_max_ = 0.985	*k* = −9→9
10616 measured reflections	*l* = −20→20

### Refinement

**Table:**

Refinement on *F*^2^	Primary atom site location: structure-invariant direct methods
Least-squares matrix: full	Secondary atom site location: difference Fourier map
*R*[*F*^2^ > 2σ(*F*^2^)] = 0.040	Hydrogen site location: inferred from neighbouring sites
*wR*(*F*^2^) = 0.101	H-atom parameters constrained
*S* = 1.02	*w* = 1/[σ^2^(*F*_o_^2^) + (0.0432*P*)^2^ + 0.2887*P*] where *P* = (*F*_o_^2^ + 2*F*_c_^2^)/3
2681 reflections	(Δ/σ)_max_ < 0.001
208 parameters	Δρ_max_ = 0.11 e Å^−^^3^
0 restraints	Δρ_min_ = −0.19 e Å^−^^3^

### Special details

**Table:**

Geometry. All e.s.d.'s (except the e.s.d. in the dihedral angle between two l.s. planes) are estimated using the full covariance matrix. The cell e.s.d.'s are taken into account individually in the estimation of e.s.d.'s in distances, angles and torsion angles; correlations between e.s.d.'s in cell parameters are only used when they are defined by crystal symmetry. An approximate (isotropic) treatment of cell e.s.d.'s is used for estimating e.s.d.'s involving l.s. planes.
Refinement. Refinement of *F*^2^ against ALL reflections. The weighted *R*-factor *wR* and goodness of fit *S* are based on *F*^2^, conventional *R*-factors *R* are based on *F*, with *F* set to zero for negative *F*^2^. The threshold expression of *F*^2^ > σ(*F*^2^) is used only for calculating *R*-factors(gt) *etc*. and is not relevant to the choice of reflections for refinement. *R*-factors based on *F*^2^ are statistically about twice as large as those based on *F*, and *R*- factors based on ALL data will be even larger.

### Fractional atomic coordinates and isotropic or equivalent isotropic displacement parameters (Å^2^)

**Table:**

	*x*	*y*	*z*	*U*_iso_*/*U*_eq_	
C1	0.37404 (14)	0.4189 (2)	0.17104 (10)	0.0410 (4)	
C2	0.32338 (16)	0.3486 (2)	0.22725 (10)	0.0485 (5)	
C3	0.20317 (17)	0.3192 (3)	0.20996 (12)	0.0563 (5)	
H3	0.1718	0.2696	0.2486	0.068*	
C4	0.13143 (16)	0.3665 (3)	0.13310 (13)	0.0540 (5)	
C5	0.17475 (16)	0.4363 (3)	0.07450 (12)	0.0554 (5)	
H5	0.1236	0.4666	0.0228	0.066*	
C6	0.29653 (15)	0.4609 (2)	0.09396 (11)	0.0463 (4)	
H6	0.3275	0.5068	0.0543	0.056*	
C7	0.50387 (15)	0.4647 (2)	0.19524 (10)	0.0455 (4)	
C8	0.57636 (13)	0.4110 (2)	0.14180 (9)	0.0380 (4)	
C9	0.54142 (14)	0.2954 (2)	0.07984 (10)	0.0394 (4)	
H9	0.4616	0.2613	0.0658	0.047*	
C10	0.60984 (14)	0.2144 (2)	0.03078 (10)	0.0371 (4)	
C11	0.54836 (15)	0.1629 (2)	−0.04971 (10)	0.0427 (4)	
H11	0.4664	0.1823	−0.0707	0.051*	
C12	0.60760 (17)	0.0835 (2)	−0.09852 (11)	0.0511 (5)	
H12	0.5661	0.0534	−0.1527	0.061*	
C13	0.72778 (18)	0.0488 (3)	−0.06717 (12)	0.0574 (5)	
H13	0.7677	−0.0051	−0.1000	0.069*	
C14	0.78932 (17)	0.0939 (3)	0.01321 (13)	0.0571 (5)	
H14	0.8703	0.0677	0.0347	0.068*	
C15	0.73151 (15)	0.1777 (2)	0.06192 (11)	0.0472 (4)	
H15	0.7740	0.2096	0.1156	0.057*	
C16	0.78369 (16)	0.4806 (3)	0.23361 (11)	0.0540 (5)	
H16	0.7819	0.4182	0.2801	0.065*	
C17	0.83462 (16)	0.6374 (3)	0.15148 (11)	0.0531 (5)	
H17	0.8810	0.7101	0.1297	0.064*	
F1	0.39533 (10)	0.30437 (19)	0.30320 (7)	0.0792 (4)	
F2	0.01212 (10)	0.3410 (2)	0.11449 (8)	0.0874 (4)	
N1	0.69278 (12)	0.48896 (18)	0.16383 (8)	0.0401 (3)	
N2	0.72498 (12)	0.5930 (2)	0.10902 (9)	0.0479 (4)	
N3	0.87605 (13)	0.5715 (2)	0.22846 (10)	0.0589 (4)	
O1	0.55016 (12)	0.5454 (2)	0.25844 (8)	0.0737 (5)	

### Atomic displacement parameters (Å^2^)

**Table:**

	*U*^11^	*U*^22^	*U*^33^	*U*^12^	*U*^13^	*U*^23^
C1	0.0413 (9)	0.0420 (10)	0.0427 (9)	0.0005 (7)	0.0173 (8)	−0.0015 (8)
C2	0.0525 (11)	0.0558 (11)	0.0415 (10)	0.0108 (9)	0.0209 (9)	0.0054 (9)
C3	0.0586 (12)	0.0590 (12)	0.0646 (13)	0.0022 (9)	0.0386 (10)	0.0056 (10)
C4	0.0387 (10)	0.0577 (12)	0.0700 (13)	−0.0016 (9)	0.0232 (9)	−0.0048 (10)
C5	0.0458 (11)	0.0632 (13)	0.0541 (11)	0.0015 (9)	0.0110 (9)	0.0039 (10)
C6	0.0463 (10)	0.0495 (11)	0.0461 (10)	−0.0032 (8)	0.0187 (8)	0.0062 (8)
C7	0.0456 (10)	0.0500 (11)	0.0404 (9)	−0.0005 (8)	0.0128 (8)	−0.0037 (8)
C8	0.0351 (9)	0.0424 (9)	0.0352 (9)	−0.0025 (7)	0.0092 (7)	0.0018 (8)
C9	0.0360 (9)	0.0425 (10)	0.0397 (9)	−0.0043 (7)	0.0119 (7)	0.0036 (8)
C10	0.0390 (9)	0.0339 (9)	0.0392 (9)	−0.0049 (7)	0.0133 (7)	0.0017 (7)
C11	0.0425 (9)	0.0406 (10)	0.0432 (9)	−0.0066 (8)	0.0107 (8)	−0.0015 (8)
C12	0.0644 (12)	0.0473 (11)	0.0429 (10)	−0.0099 (9)	0.0188 (9)	−0.0077 (8)
C13	0.0660 (14)	0.0501 (12)	0.0645 (13)	−0.0019 (9)	0.0324 (11)	−0.0123 (10)
C14	0.0443 (10)	0.0554 (12)	0.0722 (13)	0.0062 (9)	0.0192 (10)	−0.0041 (11)
C15	0.0454 (10)	0.0488 (11)	0.0443 (10)	−0.0006 (8)	0.0094 (8)	−0.0027 (8)
C16	0.0474 (11)	0.0662 (13)	0.0409 (10)	−0.0057 (9)	0.0030 (8)	−0.0005 (9)
C17	0.0427 (10)	0.0596 (12)	0.0579 (12)	−0.0127 (9)	0.0170 (9)	−0.0074 (10)
F1	0.0713 (8)	0.1213 (11)	0.0491 (7)	0.0211 (7)	0.0247 (6)	0.0255 (7)
F2	0.0441 (7)	0.1161 (11)	0.1074 (10)	−0.0115 (7)	0.0313 (7)	−0.0038 (9)
N1	0.0364 (8)	0.0457 (8)	0.0359 (7)	−0.0048 (6)	0.0079 (6)	−0.0025 (6)
N2	0.0423 (8)	0.0544 (9)	0.0476 (9)	−0.0067 (7)	0.0148 (7)	0.0024 (7)
N3	0.0430 (9)	0.0758 (12)	0.0519 (10)	−0.0112 (8)	0.0060 (7)	−0.0113 (9)
O1	0.0555 (9)	0.1069 (12)	0.0594 (9)	−0.0111 (8)	0.0190 (7)	−0.0381 (9)

### Geometric parameters (Å, °)

**Table:**

C1—C2	1.384 (2)	C10—C15	1.391 (2)
C1—C6	1.385 (2)	C10—C11	1.395 (2)
C1—C7	1.494 (2)	C11—C12	1.379 (2)
C2—F1	1.3521 (19)	C11—H11	0.9300
C2—C3	1.370 (2)	C12—C13	1.373 (3)
C3—C4	1.369 (3)	C12—H12	0.9300
C3—H3	0.9300	C13—C14	1.381 (3)
C4—F2	1.353 (2)	C13—H13	0.9300
C4—C5	1.362 (3)	C14—C15	1.381 (2)
C5—C6	1.379 (2)	C14—H14	0.9300
C5—H5	0.9300	C15—H15	0.9300
C6—H6	0.9300	C16—N3	1.312 (2)
C7—O1	1.212 (2)	C16—N1	1.332 (2)
C7—C8	1.485 (2)	C16—H16	0.9300
C8—C9	1.336 (2)	C17—N2	1.312 (2)
C8—N1	1.4302 (19)	C17—N3	1.348 (2)
C9—C10	1.464 (2)	C17—H17	0.9300
C9—H9	0.9300	N1—N2	1.3640 (19)
			
C2—C1—C6	116.68 (15)	C15—C10—C9	123.22 (15)
C2—C1—C7	121.38 (15)	C11—C10—C9	118.14 (14)
C6—C1—C7	121.63 (15)	C12—C11—C10	120.82 (16)
F1—C2—C3	117.62 (16)	C12—C11—H11	119.6
F1—C2—C1	118.97 (16)	C10—C11—H11	119.6
C3—C2—C1	123.41 (17)	C13—C12—C11	120.02 (17)
C4—C3—C2	116.88 (17)	C13—C12—H12	120.0
C4—C3—H3	121.6	C11—C12—H12	120.0
C2—C3—H3	121.6	C12—C13—C14	119.91 (17)
F2—C4—C5	118.67 (18)	C12—C13—H13	120.0
F2—C4—C3	118.26 (17)	C14—C13—H13	120.0
C5—C4—C3	123.06 (17)	C15—C14—C13	120.53 (17)
C4—C5—C6	118.19 (17)	C15—C14—H14	119.7
C4—C5—H5	120.9	C13—C14—H14	119.7
C6—C5—H5	120.9	C14—C15—C10	120.13 (16)
C5—C6—C1	121.76 (16)	C14—C15—H15	119.9
C5—C6—H6	119.1	C10—C15—H15	119.9
C1—C6—H6	119.1	N3—C16—N1	111.51 (17)
O1—C7—C8	119.98 (16)	N3—C16—H16	124.2
O1—C7—C1	120.11 (16)	N1—C16—H16	124.2
C8—C7—C1	119.91 (14)	N2—C17—N3	116.09 (17)
C9—C8—N1	120.88 (14)	N2—C17—H17	122.0
C9—C8—C7	124.84 (15)	N3—C17—H17	122.0
N1—C8—C7	114.23 (14)	C16—N1—N2	108.90 (14)
C8—C9—C10	129.73 (15)	C16—N1—C8	130.75 (15)
C8—C9—H9	115.1	N2—N1—C8	120.34 (12)
C10—C9—H9	115.1	C17—N2—N1	101.76 (14)
C15—C10—C11	118.54 (15)	C16—N3—C17	101.74 (15)
			
C6—C1—C2—F1	179.59 (16)	C7—C8—C9—C10	−170.57 (16)
C7—C1—C2—F1	−6.6 (3)	C8—C9—C10—C15	31.5 (3)
C6—C1—C2—C3	0.2 (3)	C8—C9—C10—C11	−152.16 (17)
C7—C1—C2—C3	173.98 (17)	C15—C10—C11—C12	−2.5 (2)
F1—C2—C3—C4	179.31 (17)	C9—C10—C11—C12	−179.05 (15)
C1—C2—C3—C4	−1.3 (3)	C10—C11—C12—C13	2.2 (3)
C2—C3—C4—F2	−179.28 (17)	C11—C12—C13—C14	−0.2 (3)
C2—C3—C4—C5	1.3 (3)	C12—C13—C14—C15	−1.5 (3)
F2—C4—C5—C6	−179.65 (17)	C13—C14—C15—C10	1.2 (3)
C3—C4—C5—C6	−0.3 (3)	C11—C10—C15—C14	0.8 (2)
C4—C5—C6—C1	−0.9 (3)	C9—C10—C15—C14	177.13 (16)
C2—C1—C6—C5	0.9 (3)	N3—C16—N1—N2	−0.9 (2)
C7—C1—C6—C5	−172.81 (17)	N3—C16—N1—C8	179.80 (16)
C2—C1—C7—O1	−46.0 (3)	C9—C8—N1—C16	−118.1 (2)
C6—C1—C7—O1	127.4 (2)	C7—C8—N1—C16	59.6 (2)
C2—C1—C7—C8	133.93 (18)	C9—C8—N1—N2	62.6 (2)
C6—C1—C7—C8	−52.6 (2)	C7—C8—N1—N2	−119.70 (16)
O1—C7—C8—C9	166.95 (18)	N3—C17—N2—N1	0.1 (2)
C1—C7—C8—C9	−13.0 (3)	C16—N1—N2—C17	0.46 (18)
O1—C7—C8—N1	−10.6 (2)	C8—N1—N2—C17	179.89 (15)
C1—C7—C8—N1	169.46 (14)	N1—C16—N3—C17	0.8 (2)
N1—C8—C9—C10	6.8 (3)	N2—C17—N3—C16	−0.5 (2)

### Hydrogen-bond geometry (Å, °)

**Table:**

*D*—H···*A*	*D*—H	H···*A*	*D*···*A*	*D*—H···*A*
C15—H15···N1	0.93	2.56	3.048 (2)	113
C12—H12···N3^i^	0.93	2.68	3.540 (2)	154
C17—H17···F1^ii^	0.93	2.62	3.280 (2)	128

Symmetry codes: (i) *x*−1/2, −*y*+1/2, *z*−1/2; (ii) −*x*+3/2, *y*+1/2, −*z*+1/2.
